# A 12-year follow-up of intestinal schistosomiasis in pre-school-aged children in Assoni Village, Eastern Senegal

**DOI:** 10.1186/s40249-021-00867-8

**Published:** 2021-06-27

**Authors:** Monique N’Diaye, Boubacar Fodé Keita, Fodé Danfakha, Fili Keita, Gérald Keita, Cheikh Sadibou Senghor, Bocar Diop, Lamine Diawara, François Bessin, Charlotte Vernet, Dominique Barbier, Patrick Dewavrin, Francis Klotz

**Affiliations:** 1 Normandy University, INSERM U1086 ANTICIPE (Interdisciplinary Research Unit for Cancer Prevention and Treatment), BioTICLA Axis (Biology and Innovative Therapeutics for Ovarian Cancers), Caen, France; 2 Faculty of Pharmaceutical Sciences, Department of Biodiversity Health, Microbiology, Biotechnology, UNICAEN, Caen, France; 3 NGO Le Kaïcedrat, Paris, France; 4 Hopital Principal, Dakar, Senegal; 5 Medical Center, Kedougou, Senegal; 6 Health Post, Bandafassi, Kedougou District, Dakar, Senegal; 7 Programme National de Lutte Contre Les Bilharzioses (PNLB), Ministry of Health, Dakar, Senegal; 8 Neglected Tropical Diseases, Inter-Country Support Team for West Africa, World Health Organization, Ouagadougou, Burkina Faso; 9 Military Hospital Val-de-Grâce, Paris, France

**Keywords:** Intestinal schistosomiasis, Pre-school-aged children, Praziquantel, Latrine, Prevalence, Senegal

## Abstract

**Background:**

To monitor the prevalence of schistosomiasis in school-aged children (SAC), the National Bilharzia Control Program (PNLB) was set up by the Senegalese authorities; however, geographically isolated Bedik ethnic groups that did not benefit from this program were found to be heavily infected with *Schistosoma mansoni*. This observation led us to implement a new schistosomiasis control program in 2008 under the aegis of the non-governmental organization “Le Kaïcedrat” and in partnership with the PNLB/WHO to monitor the prevalence of schistosomiasis in this area. In the village of Assoni, where 100% of SAC were infected, analysis of the stools of pre-school-aged children (PSAC) showed that they were massively infected, so we decided to focus our program on them.

**Methods:**

From 2008 to 2020, we (i) monitored the prevalence of *S. mansoni* in PSAC in Assoni using double-stool smear preparation, (ii) treated the infected PSAC with a standard dose of praziquantel 40 mg/kg, (iii) ran educational campaigns each year in the village, and (iv) built latrines to improve sanitation and reduce schistosomiasis transmission. Linear regression was used to examine the trend in the annual schistosomiasis prevalence and a two-sided of Chi-squared test was used to compare prevalence between the different age groups of PSAC.

**Results:**

We observed an extremely high prevalence of schistosomiasis (78%) in PSAC before implementation of the program in 2008. Contamination occurred in very young children, as 64.3% of children under 2 years old were infected. Moreover, prevalence increased with age and reached 96.8% in children 4 to < 6 years old. Our annual interventions in Assoni Village raised awareness among villagers that water bodies were areas of significant infestation, allowed the building of 88 latrines and led to a decrease in prevalence in PSAC as only 11% of these children were infected in 2020.

**Conclusion:**

Our study allowed Assoni to be the first village in Senegal to treat PSAC since 2014, but only on an individual basis. It also shows that schistosomiasis is difficult to eradicate and that multi-sectorial actions are required to keep its prevalence at a low level.

**Graphic abstract:**

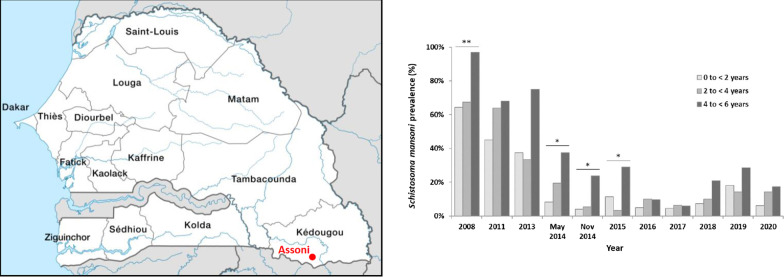

**Supplementary Information:**

The online version contains supplementary material available at 10.1186/s40249-021-00867-8.

## Background

Schistosomiasis is a human parasitic disease occurring in tropical and subtropical areas that have limited access to safe water. Almost 240 million people are estimated to be at risk of infection worldwide, 90% of whom live in Africa [1]. The disease burden in 2017 was estimated at 1.9 million disability-adjusted life-years (DALYs) and represents a major public health problem in these areas [2]. Schistosomiasis is classified as one of the main neglected tropical diseases (NTD) for which a greater global response is required to eliminate it as a public health problem [3].

Key strategies for controlling the incidence of schistosomiasis are based on multi-sectorial actions that aim to (a) block the *Schistosoma* lifecycle by preventing contamination of freshwater, removing host snails and preventing human contact with infected water bodies, and (b) treat at-risk populations with praziquantel [4, 5]. To this end, many countries in Africa have initiated large-scale control programs in partnership with the World Health Organization (WHO) by providing mass drug administration (MDA) of praziquantel for school-aged children (SAC) from 6 to 14 years old. Studies have shown that this age group is particularly infected with *Schistosoma*, since around 35% of those infected are SAC and that up to 60–80% of SAC may be infected in endemic regions [4, 6]. Of these, adolescents are the group that excretes the largest amounts of eggs [4, 7] and constitute the major reservoir of the disease [6]. Due to the limited literature on praziquantel safety and the lack of an appropriate formulation, pre-school-aged children (PSAC) have been excluded from mass treatment programs for the control of schistosomiasis [8, 9]. However, a growing number of studies have demonstrated that these children should be regarded as a high-risk group in endemic areas [8, 10–23]. These results have led to changes in the formal licensing and off-label use of praziquantel in PSAC [21]. However, until now, PSAC have been treated on a case-by-case basis and no mass drug treatment is yet authorized. Moreover, these recommendations must be accepted at the national level.

In Senegal, schistosomiasis is considered as an endemic disease and a national control program against schistosomiasis called “Programme National de Lutte contre les Bilharzioses” (PNLB—National Bilharzia Control Program) was implemented in partnership with the WHO to treat 6-to-14-year-old SAC [24]. However, several Bedik ethnic groups living in seclusion on steep hills were difficult to reach and did not benefit from the program. To improve the health network of this region and to allow these isolated populations access to care and treatment, a hospital was built in Ninefesha in 2002. In addition, the French non-governmental organization (NGO)—Le Kaïcedrat—built a parasitology laboratory and trained the technicians in detecting *Schistosoma mansoni* eggs in the stools of children from isolated villages around Ninefesha. In 2006, a preliminary study showed that 44% of the SAC in these villages were positive for *S. mansoni,* with prevalence reaching 100% in Assoni village. To perform a larger in-depth study of the prevalence of this disease around Ninefesha, an out-of-hospital program designed and funded by Le Kaïcedrat was set up in 53 Bedik and Peul villages in 2008 in agreement with the Senegalese health authorities and PNLB. The program consisted of (i) massive administration of praziquantel to more than 3000 SAC in 2008, (ii) the monitoring of schistosomiasis prevalence in these children every year, (iii) the implementation of health education campaigns and (iv) the supervision of the construction of latrines. It led to a massive fall in schistosomiasis among SAC in these territories and was followed by a stand-by program in 2015, where only villages with positive children the previous year were monitored [25].

However, given that 100% of the children had been found positive for *S. mansoni* in Assoni, we also decided to assess its prevalence in the feces of PSAC who could not benefit from praziquantel treatment until 2014. This work carried out over a 12-year period revealed that children under 6 years of age constitute a significant reservoir of *S. mansoni,* and that constant monitoring has resulted in a significant reduction in intestinal schistosomiasis in these children (78% in 2008 to 11% in 2020).

This work also allowed Assoni to become the first village in Senegal to benefit from praziquantel treatment for PSAC. It also underlined the difficulty in maintaining a low level of prevalence of *S. mansoni* and that epidemiological studies, treatments, health education campaigns and the construction of latrines, which are considered as the pillars of strategies to reduce the burden of schistosomiasis [4–6, 10, 26–28], must be constantly monitored.

## Methods

### Study design

The study was designed and financed by the French NGO “Le Kaïcedrat” and was carried out from 2008 until now with agreement from the Senegalese health authorities, the PNLB and the WHO. From 2008 to 2013, praziquantel was delivered by Ninefesha Hospital. Since the closure of the hospital in 2013, it has been delivered by the Chief Medical Officer of Kedougou District in connection with the PNLB. He also supervised the health care workers and the microscopist based at the medical post in Bandafassi.

### Assoni village

**Fig. 1:**
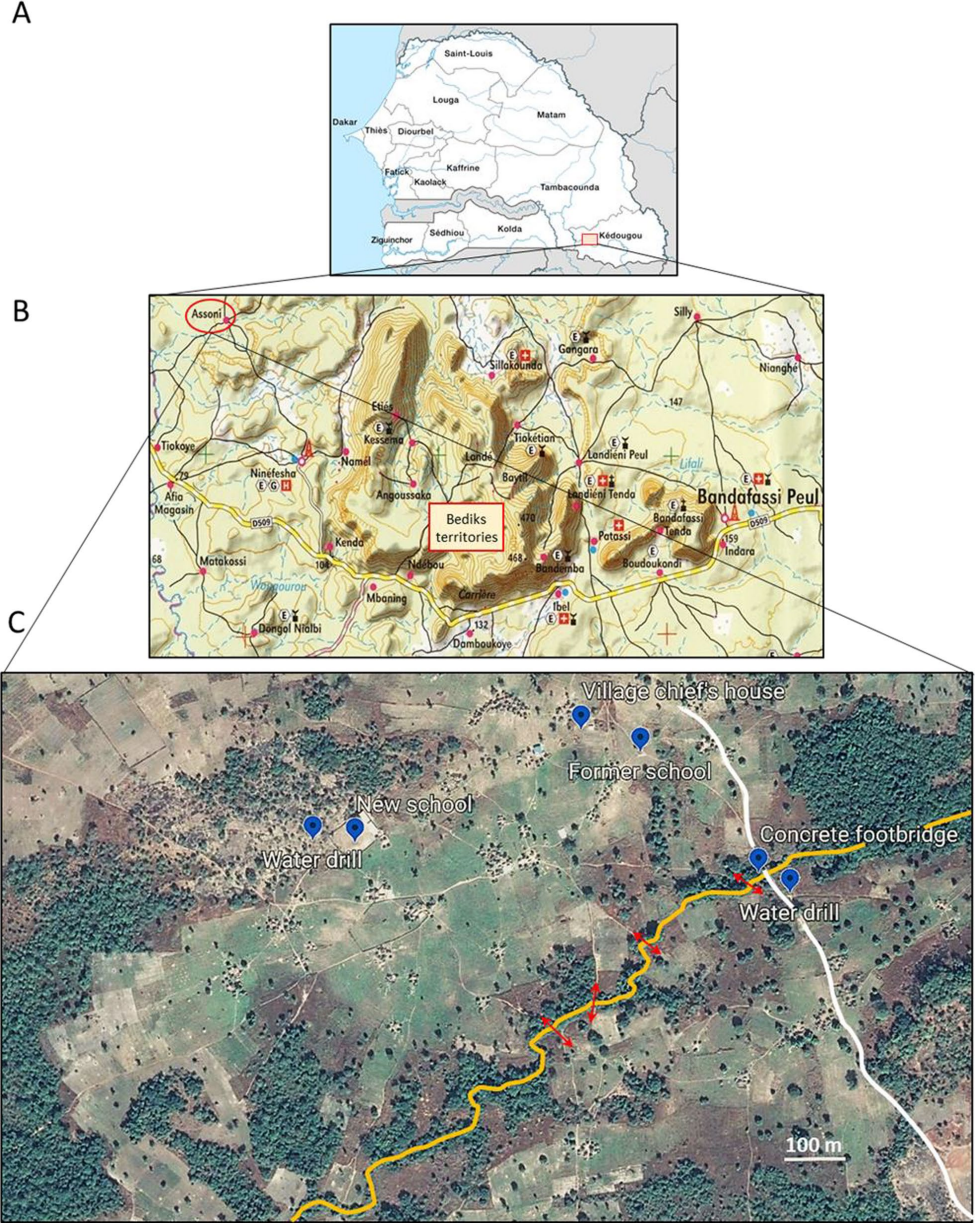
Location and topography of Assoni village. **A** Location of Assoni village in Kedougou district. **B** Location of Assoni Village and Bediks territories. **C** Assoni village is accessible by only one track which is often difficult to access (white line). It is crossed by a watercourse that turns into water bodies during the dry season (orange line). A concrete footbridge was built to allow children to go to school (former school) without crossing the backwater. However, the school was damaged and was rebuilt (new school). The many paths that join the different parts of the village and cross the infested water bodies are depicted by red arrows

Assoni is a Peul village that has 744 inhabitants and is in the South-East of Senegal in Kedougou district (Fig. 1A). It is located west of Kedougou, west of the marble hills that shelter the Bedik communities and north of Ninefesha Hospital (Latitude: 126,062,701; Longitude − 12,506,594). The Fig. 1B is based on the map ND-28-VI Kedougou (Direction des Travaux Géographiques et Cartographiques). The village is extremely spread out and does not have electricity or running water. It is crossed by a temporary watercourse which becomes a torrent during the rainy season that completely isolates Assoni from the surrounding villages. During the dry season, the river turns into water bodies that the inhabitants must cross on foot either to access the village or to reach the other side of the village. In addition, Assoni Village possesses two water drills but access to one of them requires passage through a very parasitized water body. The village also has two dug wells that dry up during the dry season. The topography of Assoni is shown in Fig. 1C that was made thanks to Google Earth software.

### Monitoring of schistosomiasis prevalence in Assoni and treatment of children

#### Parasitological examination

A technician was trained to perform a microscopic schistosomiasis diagnosis in Ninefesha Hospital. The training was funded by the NGO Le Kaïcedrat. He carried out annual monitoring of the prevalence of schistosomiasis over the 12-year period and moved to the Bandafassi post after the closure of Ninefesha Hospital in 2013. A simple smear slide was prepared for each stool sample. Stool samples were assessed at × 10 magnification by the technician. As a quality control check and to improve sensitivity, a second smear slide was performed for each stool sample.

#### Monitoring of schistosomiasis prevalence in PSAC in Assoni

A healthcare worker was trained in Ninefesha Hospital and his training as well as the motorbike required to collect stool samples from children in Assoni were funded by the NGO Le Kaïcedrat. He identified all children under 6 years old in the village and verified their name and age. An average of a hundred PSACs were registered each year in Assoni. Twenty stool samples were taken once a day on 5 consecutive days. The stools were examined by the microscopist and the number of cases of intestinal schistosomiasis in the different age groups (0 to < 2 years, 2 to < 4 years and 4 to < 6 years) was noted. It should be noted that the prevalence of schistosomiasis was monitored in 2008, 2011 and from 2013 to 2020 but not in 2009, 2010 and 2012, because some mothers refused that their children be included in the survey because they could not be treated subsequently. This issue was resolved after 2014 when Senegalese authorities accepted the use of praziquantel for PSAC.

#### Monitoring of schistosomiasis prevalence in SAC in Assoni

The mean prevalence of intestinal schistosomiasis was determined in 1/3 of all children aged 6 to 14 years chosen at random (approximately 50 children each year), attending school or not. As with PSAC, SAC were identified (name and age) and their stools were collected and analyzed. To better track positive cases, all children between 6 and 14 years old were sampled in 2019.

#### Treatment of PSAC and SAC in Assoni

PSAC could not be treated with praziquantel in Senegal until 2014. However, after 2014, thanks to the WHO authorities in Senegal, the regional medical authorities agreed to treat children under the age of 6, but only individually and with agreement from the PNLB and WHO. This authorization allowed the 16 children found positive in May 2014 to be treated. New stool samples were collected in November 2014, prevalence was assessed and infected children were treated again in December 2014. After each annual prevalence statement, the number of required praziquantel tablets was delivered by Ninefesha Hospital until 2013 and by the Kedougou District Medical Officer since 2014.

Treatment of positive cases of PSAC (40 mg/kg) was performed by a nurse who weighed the children to administer the correct dose of praziquantel. The tablet was cut and crushed in potable water and the children were kept under observation for 30 min to verify that the drug was properly absorbed. Praziquantel could be mixed with local cereal meals for children that presented difficulties to take it [23].

**Fig. 2:**
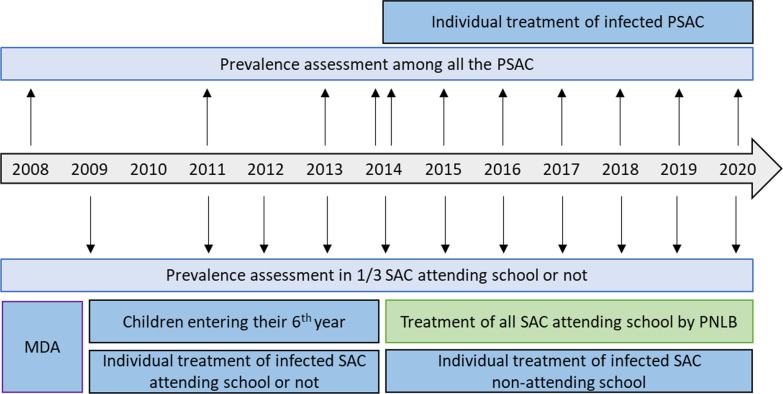
Schedule of prevalence monitoring and treatment of PSAC and SAC in Assoni village. Regarding SAC, MDA was realized in 2008 and *Schistosoma mansoni* prevalence was assessed each year in 1/3 of SAC. From 2009 to 2013, all the children entering their sixth year were treated systematically, as well as SAC who were found to be infected after prevalence monitoring. Since the closure of Ninefesha Hospital in 2013, PNLB has begun to treat SAC attending school. Non-attending school children who were found to be infected after our prevalence assessment were treated on an individual basis. In blue, prevalence assessment or treatment performed by our program; in green treatment performed by PNLB. MDA: Mass drug administration; PNLB: Programme National de Lutte contre la Bilharziose; PSAC: Pre-school-aged children, SAC: School-aged children

All SAC (aged 6 to 14 years) attending school or not were treated with praziquantel in 2008. During the following years (2009–2013), all the children entering their sixth year were treated systematically, as well as children from 6 to 14 years of age (attending school or not) who were found to be infected after prevalence monitoring. Since the closure of Ninefesha Hospital in July 2013 and as it was extremely difficult to reach some villages, the children who could easily be supervised, i.e. those aged 6 to 14 years attending school who comprised 98% of the children in this age group, were treated by PNLB during its annual Mass Drug Administration campaign. Children not attending school who we found to be infected after our prevalence assessment were treated on an individual basis by a healthcare worker, who also verified that the drug was well absorbed. The schedule of PSAC and SAC prevalence monitoring and treatment is shown in Fig. 2.

### Health education campaigns

#### Schistosomiasis lifecycle illustrations

The educational campaigns were carried out each year from 2008 to 2020. During meetings with the villagers, our team used card games depicting the parasite's life cycle, short video reports of how the *Schistosoma* eggs hatch and freshwater snails collected from the contaminated sites. This part of the health education campaign was designed to bring as many villagers as possible together with the civil and religious leaders to make sure everyone was aware of the risks. We placed special emphasis on explaining to mothers how their children could be infected, e.g. by becoming infected while bathing in ponds. To this end, a few days before each health education campaign starts, the village chief is informed and we wait until all the mothers can attend the meeting in order to talk with the greatest number.

#### Building of latrines

The construction of pit latrines is the easiest way to interrupt the life cycle of the parasite. Our design was a pit measuring 1.8 m × 1.8 m × 2.5 m that was covered with a reinforced concrete slab poured by a mason. The villagers were paid to dig the pit, but only after building and installing a fence around the latrine, allowing it to be used day and night. The financing of these latrines came from donations (100 EUR per latrine) and comprised the price of the materials, the rent of a truck to bring the materials to Assoni, and the cost of the bricklayer and the villagers.

### Data analysis

Annual schistosomiasis prevalence was presented as a percentage of the number of children infected in an age group out of the total number of children in the same age group. Linear regression was used to examine the trend in the annual schistosomiasis prevalence from the beginning of the study to 2020. A two-sided of Chi-squared test was used to compare prevalence between the different age groups of PSAC. Results were considered statistically different if *P* < 0.05 (*); *P* < 0.01 (**); *P* < 0.001 (***). Data were entered using Microsoft Excel® spreadsheet software. Statistical analyses were conducted using R studio software [R Core Team (2019). R: A language and environment for statistical computing. R Foundation for Statistical. Computing, Vienna, Austria. https://www.R-project.org/].

### Ethical guidelines

The study protocol was approved by the WHO Neglected Tropical Diseases Inter-Country Support Team for West Africa, PNLB, Health and Social Action Ministry in Senegal. At the beginning of the study, the regional director of health, the village authorities, and local community members were fully informed about the objectives of the project. In details, a community-based approach was used and, in this regard, we met the local traditional community leaders to explain the aim of the study and the necessity to collect inhabitants’ stool samples to realize a study for public health improvement. After their agreements, we were authorized to get in touch with the population to present the latrines construction project. For the people who agreed to participate to the study, we asked for stool samples to evaluate the prevalence of *S. mansoni* in the village and to assess if the actions we carried out (i.e. the building of latrines and praziquantel treatment) were effective.

## Results

### Prevalence of intestinal schistosomiasis in PSAC in Assoni

Since children were not allowed to be treated with praziquantel until 2014, the only way to reduce the prevalence of schistosomiasis was to avoid contamination via the water bodies.

**Fig. 3:**
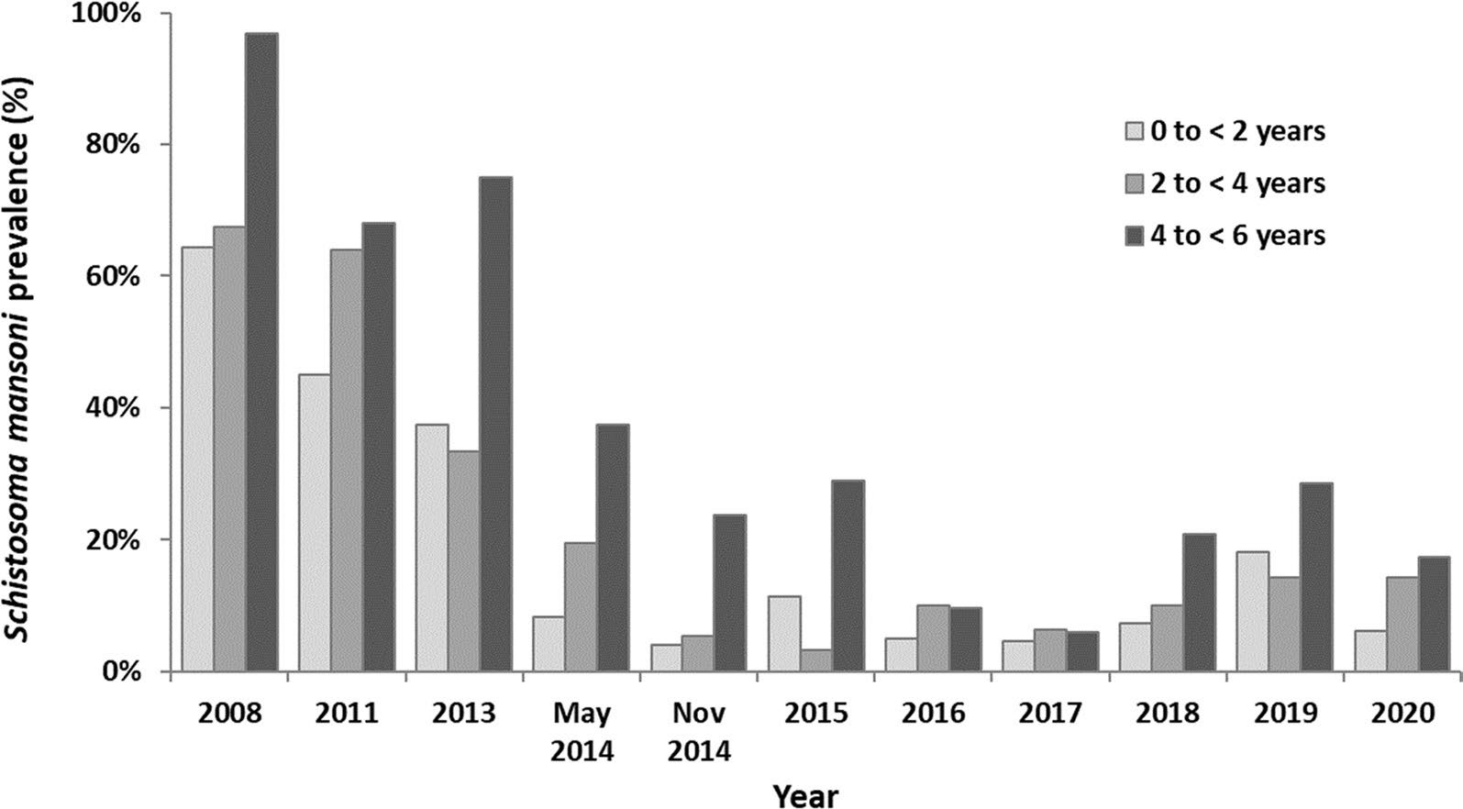
Prevalence of *Schistosoma mansoni* among different age groups of pre-school-aged children (PSAC) in Assoni. Prevalence in PSAC was assessed at 0 to < 2, 2 to < 4 and 4 to < 6 years of age from 2008 to 2020. Prevalence was not assessed in 2009, 2010 and 2012 because mothers did not want their children to be sampled as they could not benefit from any treatment

Cases of *S. mansoni* infestation were reported in the PSAC of Assoni from 2008 to 2020 (Fig. 3 and Additional file 1). The prevalence observed in 2008 revealed that the three age groups were massively infested before the program has been implemented. Briefly, 96.8% of children aged 4 to < 6 (30/31 children), 67.5% of children aged 2 to < 4 (25/37 children) and even 64.3% of children under 2 years (9/14 children) were contaminated. Based on the results, children aged 4 to < 6 appeared to be the most infected (*P* = 0.0059).

After the yearly health education campaigns, the prevalence dropped drastically in the three age groups until May 2014 [8.3% (3/36) for children aged 0 to < 2 years, 19.4% (7/36) for children aged 2 to < 4 years, and 37.5% (6/16) for children aged 4 to < 6 years (who were found statistically more infected, *P* = 0.0458)]. The results showed that among the 83 PSAC living in Assoni, 8 (10%) were still infected with *S. mansoni*. However, this result masked some discrepancies. Indeed, whereas prevalence dropped to 4.0% (1/25) and 5.4% (2/37) for children aged 0 to < 2 and 2 to < 4 years, it remained around 23.8% (5/21) for children aged 4 to < 6 years (*P* = 0.0385). Moreover, 2 children between 4 and 6 years old already found positive and treated in May were re-infected between May and November 2014, suggesting that advice given to mothers during educational health campaigns was not followed in certain families.

Although the prevalence of intestinal schistosomiasis slightly increased in 2015 [11.3% (6/53), 3.2% (1/31) and 29.1% (7/24) for children aged 0 to < 2 years, 2 to < 4 years and 4 to < 6 years respectively – the latter group being more infected than the other groups, *P* = 0.0156], the prevalence in PSAC still decreased until 2017 to reach its lowest level (5.4% on average in the three age groups) with 4.5% (2/44), 6.5% (2/31) and 5.9% (1/17) for children aged 0 to < 2 years, 2 to < 4 years and 4 to < 6 years. The results showed that the decline was particularly notable in children aged 4 to < 6 years (29.1% in 2015, 9.7% in 2016 and 5.9% in 2017).

After this encouraging result, a rebound occurred in 2018 and 2019 with an increase in the prevalence in the three groups of PSAC [18.2% (6/33), 14.3% (5/35) and 28.6% (6/21) for children aged 0 to < 2 years, 2 to < 4 years and 4 to < 6 years respectively], showing that almost a third of the children aged 4 to < 6 years were found to be infected in 2019.

In December 2020, prevalence stabilized at 11.8% [6.3% (2/32), 14.3% (3/21) and 17.4% (4/23) for children aged 0 to < 2 years, 2 to < 4 years and 4 to < 6 years respectively]. All positive cases were reported annually and treated individually, as described in the Methods section. Note that all the positive cases found after 2014 were new cases and not cases of re-infestation.

### Average intestinal schistosomiasis prevalence in SAC in Assoni

**Fig. 4:**
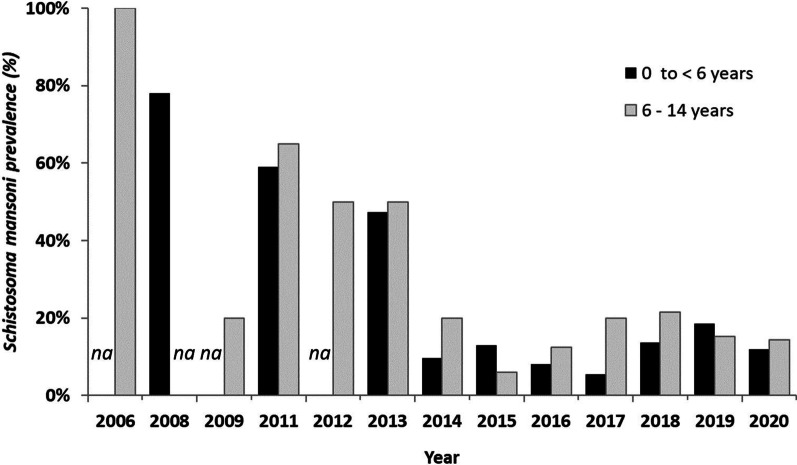
Comparison between prevalence of *Schistosoma mansoni* among pre-school-aged children (PSAC) and school-aged children (SAC) in Assoni. *Schistosoma mansoni* prevalence was assessed in Assoni from 2006 to 2020 in PSAC and SAC. *na: no available data*

After the MDA carried out by our program in 2008 in agreement with the PNLB and the WHO, prevalence that was 100% (42/42) in 2006 fell to 20% in 2009 (4/20), demonstrating the effectiveness of the treatment (Fig. 4 and Additional file 1). However, it rebounded sharply in 2011 to 65% (26/40) and decreased dramatically until its lowest level (6%, 3/50) in 2015. Although a notable rebound was observed in 2017 and 2018 (> 20%, 9/42), the prevalence at present remains low around 14% (7/79).

It is noteworthy that the prevalence in the two age groups (SAC and PSAC) followed the same evolution from 2011: a large decrease followed by a slight rebound that seemed to be stabilized in 2020 (the average prevalence between 2015 and 2020 was 14.9% ± 4.5% in SAC and 11.7% ± 4.5% in PSAC). The decrease of schistosomiasis prevalence observed in the 2 age groups from the beginning of the study to 2020 was statistically significant (*P* = 0.0015 for 0 to < 6 years and *P* = 0.0045 for 6 to 14 years).

### Schistosomiasis lifecycle illustrations

The meetings with the villagers offer precious opportunities for them to learn about the life cycle of *Schistosoma*. In addition, practical advice is provided so that the residents avoid contaminating themselves. Mothers represent the target audience as they perform the majority of domestic work and care for young children [10, 11, 17, 19, 33, 34, 44]. The explanations were translated in Peul dialect. Lifecycle illustrations, videos showing eggs hatching and snail specimen were presented to villagers who were very attentive and interested. They were informed of the release of the cercariae, the infesting forms, in the water bodies. Villagers also explained the importance of avoiding backwater to those who did not understand. At the end of the meeting, a summary highlighting the key elements of the mode of contamination was realized and we asked the active participation of the villagers to be sure that the whole discussion was understood.

### Building and use of latrines by inhabitants

There were no latrines in the village before the program. As shown in Table 1, from 2008 to 2020, 88 latrines were built. Actually, the village was gradually equipped with latrines because funding was obtained progressively. In addition, every year the damaged latrines were identified and repaired as soon as possible. Thus in 2020, 74 latrines are functional and 14 damaged latrines have been rebuilt in this village. The rate reaches one latrine per 10 inhabitants, thanks to the inhabitants' keen interest in improving their health and comfort.

**Table 1:** Number of latrines built each year in Assoni

Years	Latrines		
	New	Damaged	Rebuilt
2008	0	0	0
2009	**16**	0	0
2010	**45**	0	0
2011	0	0	0
2012	0	0	0
2013	0	0	0
2014	**4**	0	0
2015	0	**4**	0
2016	0	0	0
2017	**4**	**6**	**6**
2018	0	0	0
2019	**4**	**8**	**8**
2020	**5**	0	0
**Total**	**78**	**18**	**14**
**Total functionnal latrines**	**74**		

Latrine building was supervised by a healthcare worker. Each latrine was funded by donations explaining why the village was equipped progressively. Each year, damaged latrines were identified and rebuilt as soon as possible

## Discussion

Intestinal schistosomiasis is an insidious neglected tropical disease and is a chronic public health problem in Africa. The results of our previous study showed that *S. mansoni* and *S. haematobium* are endemic in ethnic group villages around Ninefesha and that a large percentage of children was infected [25]. Nonetheless, our program led to a significative drop in schistosomiasis prevalence in all the 53 villages. As 100% of the children were positive for *S. mansoni* in Assoni, it became our sentinel village and samples from PSAC were tested in parallel with those of SAC to assess the prevalence of the disease in the village. Whereas SAC could benefit from PNLB treatment, a clear “praziquantel treatment gap” became apparent with PSAC, as this drug is not licensed for children under 6 years old. The reasons are complex and include the absence of appropriate pediatric formulation, the uncertainties in levels of exposure to infected water sources in this age group, and the uncertain safety profile of praziquantel in children [8, 9]. The WHO provided new evidence that these children should be regarded as a high-risk group in endemic areas and encouraged changes in praziquantel formal licensing or off-label use in the treatment of PSAC [21]. For this purpose, infected PSAC should be provided with crushed tablets of praziquantel, a treatment that has been demonstrated to be safe for this age group [4, 8, 9, 11, 14, 18, 19, 21, 29–32]. The investigation began in Assoni before 2014, i.e. before praziquantel treatment was authorized for this age group in Senegal.

### *S. mansoni* prevalence among PSAC in Assoni

The average prevalence rate we found in 2008 reached 78% and rose to 100% in children aged from 4 to < 6 years old. This result shows that contamination occurred before 2 years of age, a finding supported by several studies showing that active infection can occur in the very young [4, 9–11, 13–16, 18–20, 31, 33–35]. Moreover, prevalence was maximal (96.8%) in children aged from 4 to < 6 years old, which is in concordance with the prevalence of 100% found in SAC in 2008 and with other studies showing massive infestation in SAC [13, 14, 16, 18, 20, 36–38]. Until 2014, no treatment was authorized for PSAC and the sharp decrease in *S. mansoni* prevalence (from 78% in 2008 to 18% in May 2014) reflects the success of our annual health educational campaigns and the building of latrines.

Thanks to the WHO authorities in Senegal, the medical regional authorities accepted to treat children under 6 years old but only on an individual basis and under PNLB supervision in 2014. To our knowledge, Assoni was the first village in Senegal to treat PSAC children. Since then, the overall prevalence in PSAC has been around 11%, despite fluctuations in 2018 and 2019. This result is due to the constant control of prevalence, the use of latrines and individual treatments. It constitutes the marker of the success of our program as no mass treatment program exists for these children.

### Comparison of prevalence in PSAC and SAC between 2009 and 2020

After the encouraging result in 2009 (20%), the prevalence of schistosomiasis in SAC strongly increased again, so the advice given during the educational campaigns had not been followed properly. The re-emergence of schistosomiasis after treatment in highly endemic areas has previously been reported in Ivory Coast and Niger [39, 40]. An increase in *S. mansoni* prevalence also occurred in Senegal 10 months after praziquantel administration, in Mali after four rounds of praziquantel treatment and in Ivory Coast one year after MDA [27, 36, 41]. This could be due to the fact that praziquantel does not kill immature *Schistosoma* and cannot prevent reinfection, so its efficacy has only a temporary effect on transmission and treatment has to be backed up by constant health education campaigns [42]. Moreover, previous studies showed that the efficacy of praziquantel was undermined in the event of massive infestation [40, 43].

As PSAC have never received MDA, this result proves that complementary interventions such as water sanitation and hygiene (WASH) and information, education and communication (IEC) play a major role in morbidity control, as shown by other studies [4–6, 8, 11, 12, 16, 17, 19, 26, 33, 34, 37, 44, 45]. Interestingly, the rebound occurred earlier in children from 6 to 14 years old (2016) than in those from 0 to 5 years old (2018), suggesting that understanding of the educational health campaigns, the use of latrines and backwater avoidance is more difficult to instill in SAC, who are perhaps more careless and come more readily into contact with infested water than their mothers who look after PSAC [18]. Another hypothesis that could explain this rebound is the major role played by ecological and seasonal factors in schistosomiasis re-infection/incidence after successful treatment. In fact, long rainy seasons may fill up infested backwater ponds and streams and increase the prevalence of schistosomiasis [6, 36, 37, 45, 46].

An obvious caveat for our data is the method used for diagnosing the prevalence of *S. mansoni*. Double direct observation of stool smears lacks sensitivity, especially when the prevalence drops [35, 38]. Moreover, the intensity of infestation and the efficacy of treatment were not assessed, which undermines the accuracy of our data. Importantly, the Assoni and Bedik territories are very difficult places to reach. In these remote areas, direct observation of fecal smears under a solar microscope was the only technique that could be carried out routinely, since the post at Bandafassi had no running water or electricity until 2020. Sousa-Figueiredo et al. suggested that routine egg observation from stool samples could be an appropriate technology in remote high-prevalence areas to estimate the local prevalence of intestinal schistosomiasis [18]. Furthermore, the same well-trained microscopist performed all the observations since the beginning of the program in 2006, thus ensuring continuity in diagnostic quality.

### Latrines

Given the high prevalence of schistosomiasis in this area, latrines were required as a means complementary to educational campaigns and treatments to control schistosomiasis. To be effective, they had to be accepted by the population. Latrines have been shown to avoid water body contamination and to be a useful adjunct to treatment and education in schistosomiasis transmission [18, 21, 28, 31, 32]. When the conditions are met, they prove to be used frequently and to be kept clean, and the villagers seem to appreciate the improved comfort they offer and recognize them as a measure to preserve the health of their children (fewer children with 'big bellies'). They are willing to cooperate fully and demand on their part is unanimously high. Most of the latrines in Assoni were built in 2010, a year coinciding with the beginning of the decrease in *S. mansoni* prevalence. Moreover, our yearly visits show that the latrines are properly maintained and remain functional in the long term because only the oldest have needed rebuilding, and they have resisted the harsh rainy seasons when floods regularly damage the villagers’ homes and isolate them from the outside world.

### 12-year follow-up and difficulties encountered

To our knowledge, this 12-year study is the first to investigate the prevalence of *S. mansoni* over such a long period. We show that the elimination of schistosomiasis is extremely difficult and that three major parameters have to be constantly supervised to control morbidity.

The first parameter concerns the collection of samples to evaluate prevalence. It was very difficult to collect stool samples from PSAC especially before 2014, as they were not allowed to be treated. Mothers refused to allow their children to participate in the program because no treatment could be administered if they were found to be infected. Thus, as explained in Methods section, we were unable to collect PSAC stool samples in 2009, 2010 and 2012. After 2014, this problem was resolved. Moreover, as we have returned every year for 12 years, a relationship of trust has been established with the inhabitants and mothers are now less reluctant to allow their children to participate in the program because they realize the benefit for them. While collecting stool samples from children under 2 years old was difficult to organize and perform, it showed that contamination occurs from early infancy, which justifies the need for these children to enter a treatment program for schistosomiasis.

The second point explaining the difficulty in eradicating schistosomiasis is the problem of the follow-up of children. Concerning PSAC, no noticeable difficultly was encountered and the mothers generally allowed the healthcare worker to treat their children. A single dose treatment was administered, as advocated by the WHO and other studies which demonstrated that this regimen is effective in PSAC [14, 32]. The healthcare worker supervised the administration to be sure that the children took praziquantel correctly. No adverse effect was noted, as also observed in other studies [8, 14, 18, 19, 21, 30, 31, 47].

However, as there were too many SAC between 6 and 14 years old to monitor, only 1/3 chosen at random were sampled each year, which could have caused a lack of precision and led to some positive cases not being treated. To offset this difficulty, all children between 6 and 14 years old were tested in 2019. However, the prevalence remained the same around 15%, suggesting new contaminations and a lack of compliance with advice provided during the health education campaigns. Of note, every year since 2014, the PNLB has treated SAC but only those who can be easily supervised, i.e. those attending school. In consequence, children who do not go to school are not offered treatment and their mobility could promote the spread of the disease. Indeed, studies have shown that non-school-attending SAC have similar or higher prevalence rates of infections compared to school-attending children [38, 48]. These data encourage us to continue to work in parallel with the PNLB in order to track non-school-attending infected children and to better control the disease. Moreover, as praziquantel is now administered by a teacher and not by a healthcare worker who is more aware of the serious risks of schistosomiasis, this could lead to a lack of compliance from the children so praziquantel administration should be careful monitored, as also suggested in previous studies [27, 37].

To counter the potential bias due to the treatment provided by the PNLB, we evaluated the prevalence of schistosomiasis before their arrival in the village to evaluate the eventual re-infestations in SAC. Coordination with PNLB is therefore essential: for the population, as we track children who have not been treated or have been re-infected; and for the PNLB, as it could benefit from our results on schistosomiasis prevalence in order to focus treatment on villages that present recurrent infestation.

The third pitfall is the difficulty in improving health education. Even if the inhabitants tried to change their behavior, some circumstances have posed a problem for them. For example, Assoni is a very large village that covers a great distance along a backwater that villagers have to cross to reach the other part of the village. The constant round trips through water bodies could explain the difficulty in eradicating schistosomiasis. To this end, a large concrete footbridge was built in 2010 at the entrance to the village to allow access to the school and thus bypass the infested waters. However, the school, which was initially located near the footbridge, was destroyed during the rainy season and has been moved 500 m to the West, so the only footbridge that allowed the backwater to be avoided is no longer on the way to school; so the children continue to cross the infested waters.

As far as the educational campaigns are concerned, it is sometimes difficult to bring everyone together. Some mothers have not regularly attended these health education meetings, either because they did not dare to participate, or were working in the fields or looking after the livestock. In turn, this promotes the passive exposure to infection of PSAC when their mothers perform daily water-related chores in the infested waters. This could partly explain the difficulty of eliminating the disease in PSAC [18, 27].

Another important point is that the Senegalese population is very mobile so children who do not go to school travel a lot between the different villages to see their relatives. This leads them to be in contact with treated children, to indirectly infect them and thereby spread the disease [27].

A final concern is the putative involvement of an animal reservoir. Several studies have shown that non-human primates as well as rodents likely play the role of host in the *S. mansoni* cycle [49–53]. The maintenance of a reservoir sylvatic cycle could be a potential ecological cause of human reinfection that would undermine the results of schistosomiasis control programs. This underlines the importance of undertaking cross-disciplinary interventions as well as long-term schistosomiasis prevalence control to guide preventive measures.

In summary, the many difficulties encountered during these 12 years led to several limitations in the study. These limitations concerned prevalence assessment (sample collection, technical method) follow-up of children treatment (mobile population, praziquantel administration monitoring) and health education campaigns (behavioral drifts). However, despites these pitfalls, our yearly monitoring and building of latrines led to a strong reduction of intestinal schistosomiasis transmission.

## Conclusions

Altogether, our results show that the geographically isolated village of Assoni is a newly discovered site where intestinal schistosomiasis is rife. The high prevalence of schistosomiasis in PSAC in Assoni highlights the fact that they are an important reservoir and have to be treated to reduce disease transmission as well as chronic sequelae. As efforts are underway to move from morbidity control to elimination, chemotherapy might need to be expanded to this age group. Our results also show that WASH and information, education and communication (IEC) play a major role in morbidity control, as supported by other studies. These points are all the more important as several studies point to a possible reduced efficacy of praziquantel in the future due to large-scale treatments carried out every year. This 12-year study demonstrates that these issues have to be constantly addressed, that the task is thankless, and that the desired goals are difficult to attain especially in areas of high transmission. Nevertheless, the medical community cannot sidestep them if the prevalence of schistosomiasis is to be controlled in the long term.

## Supplementary Information

**Additional file 1.** Prevalence of *Schistosoma mansoni* among PSAC and SAC in Assoni (raw data). *Schistosoma mansoni* prevalence was assessed in Assoni from 2008 to 2020 in PSAC and SAC.

## Data Availability

Data supporting the findings of this study are available on reasonable request from the corresponding author [MN]. The data are not publicly available because the study is part of a larger project (https://kaicedrat.org/nos_actions/programme-bilharziose/) that is still ongoing.

## Abbreviations

IEC: Information, Education and Communication
MDA: Mass drug administration
NTD: Neglected Tropical Disease
PSAC: Pre-school-aged children
PNLB: Programme National de Lutte contre la Bilharziose
SAC: School-aged children
WASH: Water sanitation and hygiene
WHO: World Health Organization
