# Supplementary material for: A 12-year follow-up of intestinal schistosomiasis in pre-school-aged children in Assoni Village, Eastern Senegal
Source: Infect Dis Poverty. 2021 Jun 27;10:89. doi: 10.1186/s40249-021-00867-8 (PMC8237420; doi:10.1186/s40249-021-00867-8)
Supplement: Supplementary file 1 — Additional file 1. Prevalence of Schistosoma mansoni among PSAC and SAC in Assoni (raw data). Schistosoma mansoni prevalence was assessed in Assoni from 2008 to 2020 in PSAC and SAC. [file 40249_2021_867_MOESM1_ESM.pdf]

Year	Prevalence	Age groups				
		0 - <2 years	2 - <4 years	4 - < 6 years	**0 - < 6 years**	**6 - 14 years**
2006	P / T	*na*				**42/42**
	%	*na*				**100**
2008	P / T	9/14	25/37	30/31	**64/82**	*na*
	%	64,3	67,5	96,8	**78**	*na*
2009	P / T	*na*				**4/20**
	%	*na*				**20**
2011	P / T	5/11	18/28	15/22	**42/61**	**26/40**
	%	45	64	68	**59**	**65**
2012	P / T	*na*				**20/40**
	%	*na*				**50**
2013	P / T	3/8	6/18	9/12	**18/38**	**20/40**
	%	37,5	33,3	75	**47,4**	**50**
May 2014	P / T	3/36	7/36	6/16	**16/88**	*na*
	%	8,3	19,4	37,5	**18,2**	*na*
November 2014	P / T	1/25	2/37	5/21	**8/83**	**8/40**
	%	4	5,4	23,8	**9,6**	**20**
2015	P / T	6/53	1/31	7/24	**14/108**	**3/50**
	%	11,3	3,2	29	**12,9**	**6**
2016	P / T	2/37	2/20	4/41	**8/98**	**5/40**
	%	5	10	9,7	**8,2**	**12,5**
2017	P / T	2/44	2/31	1/17	**5/92**	**9/45**
	%	4,54	6,45	5,88	**5,4**	**20**
2018	P / T	2/27	4/40	9/43	**15/110**	**9/42**
	%	7,4	10	20,9	**13,6**	**21,4**
2019	P / T	6/33	5/35	6/21	**17/92**	**23/151**
	%	18,2	14,3	28,6	**18,5**	**15,2**
2020	P / T	2/32	3/21	4/23	**9/76**	**7/49**
	%	6,25	14,3	17,4	**11,8**	**14,3**
